# Type 1 diabetes impairs the activity of rat testicular somatic and germ cells through NRF2/NLRP3 pathway-mediated oxidative stress

**DOI:** 10.3389/fendo.2024.1399256

**Published:** 2024-05-16

**Authors:** Massimo Venditti, Maria Zelinda Romano, Serena Boccella, Asma Haddadi, Alessandra Biasi, Sabatino Maione, Sergio Minucci

**Affiliations:** ^1^ Dipartimento di Medicina Sperimentale, Università degli Studi della Campania “Luigi Vanvitelli”, Napoli, Italy; ^2^ Laboratoire LR11ES41 Génétique Biodiversité et Valorisation des Bio-Ressourcés Institut Supérieur de Biotechnologie de Monastir, Université de Monastir, Monastir, Tunisia

**Keywords:** spermatogenesis, steroidogenesis, blood-testis barrier, INSL3, RXFP2, SIRT1, inflammasome, apoptosis

## Abstract

**Background:**

It is well known that metabolic disorders, including type 1 diabetes (T1D), are often associated with reduced male fertility, mainly increasing oxidative stress and impairing the hypothalamus–pituitary–testis (HPT) axis, with consequently altered spermatogenesis and reduced sperm parameters. Herein, using a rat model of T1D obtained by treatment with streptozotocin (STZ), we analyzed several parameters of testicular activity.

**Methods:**

A total of 10 adult male Wistar rats were divided into two groups of five: control and T1D, obtained with a single intraperitoneal injection of STZ. After 3 months, the rats were anesthetized and sacrificed; one testis was stored at -80°C for biochemical analysis, and the other was fixed for histological and immunofluorescence analysis.

**Results:**

The data confirmed that T1D induced oxidative stress and, consequently, alterations in both testicular somatic and germ cells. This aspect was highlighted by enhanced apoptosis, altered steroidogenesis and Leydig cell maturity, and impaired spermatogenesis. In addition, the blood–testis barrier integrity was compromised, as shown by the reduced levels of structural proteins (N-cadherin, ZO-1, occludin, connexin 43, and VANGL2) and the phosphorylation status of regulative kinases (Src and FAK). Mechanistically, the dysregulation of the SIRT1/NRF2/MAPKs signaling pathways was proven, particularly the reduced nuclear translocation of NRF2, affecting its ability to induce the transcription of genes encoding for antioxidant enzymes. Finally, the stimulation of testicular inflammation and pyroptosis was also confirmed, as highlighted by the increased levels of some markers, such as NF-κB and NLRP3.

**Conclusion:**

The combined data allowed us to confirm that T1D has detrimental effects on rat testicular activity. Moreover, a better comprehension of the molecular mechanisms underlying the association between metabolic disorders and male fertility could help to identify novel targets to prevent and treat fertility disorders related to T1D.

## Introduction

Reproduction is an essential feature of all living organisms, as it allows them to produce fertile offspring, ensuring the survival of their species. A key event of this process is gametogenesis, which is comprehensive of the development and maturation of specialized cells, spermatozoa (SPZ), and oocytes. During gametogenesis, germ cells (GC) go through diverse stages like mitosis, meiosis, and differentiation, and the whole progression is finely and complexly regulated by a multitude of elements, including the expression of stage-specific genes and hormones produced by the hypothalamus–pituitary–gonads axis as well as local signaling modulators between the somatic and germinal compartments (1).

Due to the intrinsic complexity underlying reproductive homeostasis, and the countless factors involved, gametogenesis may be quite easily compromised, drastically lowering gamete quality and their ability to fertilize/be fertilized. Disorders in gametogenesis can lead to subfertility/infertility, which is a condition that affects a significant portion of the global population. Male infertility accounts for approximately 50% of the cases (2, 3), and it is mainly manifested with poor-quality sperm in terms of quantity (oligozoospermia), morphology (globozoospermia), and motility (asthenozoospermia) (4, 5).

Although, on one hand, approximately 30% of male infertility cases are still considered idiopathic, a wide range of factors can be counted among the known causes of reduced fertility (6), including genetic and hormonal abnormalities, exposure to environmental pollutants (including heavy metals, endocrine disrupting chemicals, and microplastics) (7, 8), lifestyle (smoking, alcohol consumption, and overweight/obesity) (9), and different disease, including cancer (10), varicocele (11), and metabolic disorders (12, 13).

Among the latter, diabetes mellitus (DM) is one of the most threatening due to its meaningful association with mortality and morbidity (14). DM, a chronic disease characterized by hyperglycemia consequent to insulin deficiency (type 1 diabetes, T1D) and/or insulin resistance (type 2 diabetes), is often associated with metabolic syndrome (15). Indeed DM is characterized by several manifestations, such as dyslipidemia, hypertension, oxidative stress, chronic inflammation, mitochondrial dysfunction, and endoplasmic reticulum stress.

DM, as well as other metabolic disorders, including obesity (16–19), mainly acts via two principal mechanisms: increased oxidative stress and impaired hypothalamus–pituitary–testis (HPT) axis, which has been associated with the increasing rates of male sub-infertility by provoking damage in testicular somatic (Leydig and Sertoli cells) and GC, with consequently altered spermatogenesis and reduced sperm parameters (20). In particular, the hypoglycemic state is mainly responsible for reactive oxygen species (ROS) hyperproduction and, consequently, oxidative stress via the generation of hydroxyl radicals by the autoxidation of glucose as well as the overproduction of advanced glycation end products created by nonenzymatic reactions between sugar and amino groups of proteins (21). It has been well recognized that oxidative stress is one of the main causes of infertility, independently of its source (22), since it induces testicular germ and somatic cells to produce excessive ROS, overwhelming the endogenous ROS scavenging systems and causing damage to many cellular macromolecules and organelles (21). In this regard, SPZ are particularly susceptible to oxidative damage because of the considerable amount of polyunsaturated fatty acids (PUFA) in their plasma membrane and the low concentration of antioxidant enzymes and DNA repair system in the cytoplasm and nucleus, respectively, leading to a significant decrease in sperm count and semen quality (21).

The major impact of oxidative stress on fertility has also been highlighted by the fact that many intervention strategies to face infertility are just addressed to reduce oxidative stress—for example, using antioxidant molecules (23–31). In this regard, our recent research focuses on the cellular and differentiative mechanisms that regulate spermatogenesis, which was perturbed using experimental models of oxidative stress (32, 33), to expand the knowledge of the molecular pathways involved in spermatogenesis.

In this paper, using a rat model of T1D obtained by treatment with streptozotocin (STZ), we confirmed previous works (reviewed in (20, 34)) and evaluated several additional parameters of testicular activity. We focused the attention on both the somatic and germinal compartments of the testis since Leydig and Sertoli cells’ dysfunction was assessed via the analysis of steroidogenesis and Leydig cell (LC) maturity using, as a marker, the insulin-like factor 3 (INSL3)–relaxin family peptide receptor 2 (RXFP2) system as well as the blood–testis barrier (BTB) integrity. Mechanistically, because many reports demonstrated the association of SIRT1 (35, 36), NRF2 (37, 38), MAPKs (39, 40), and inflammasome (41) pathways with cellular function altered by oxidative stress, we further verified whether these pathways may also be involved in the molecular mechanisms underlying the T1D rat testicular dysfunction.

## Materials and methods

### Animals, treatment, and sample collection

Two-month-old male Wistar rats (*Rattus norvegicus*, *n* = 10), weighing 220 ± 18.97 g, were acclimated in individual stainless steel cages under controlled conditions of light (12-h light and 12-h dark cycles), temperature (24 ± 2°C), and humidity (55 ± 20%) for 1 week before the beginning of the experiments. The animals had free access to food and water *ad libitum*. The rats were divided into two groups: the control (C; *n* = 5) receiving 5% citrate buffer solution (#211018; AppliChem GmbH, Darmstadt, Germany) and the treated group (T1D; *n* = 5) which received a single i.p. injection (65 mg/kg body weight) of STZ (#18883–66-4; Chem Cruz Biochemicals; Huissen, Netherlands). The STZ solution was freshly prepared in a cold 0.1-M citrate buffer (pH = 4.5). Body weight and serum glucose levels were assessed before STZ administration (day 0) and once per week until the end of the experimental observations. Measurements of glucose concentration were obtained from whole blood samples taken from the rat tail vein. The parameter, expressed in milligrams per deciliter, was determined by using a standard clinical blood glucometer set for testing glycemia (#GlucoMen LX2; A. Menarini Diagnostics, Winnersh, United Kingdom).

After 3 months, all the rats were anesthetized by an i.p. injection of chloral hydrate (#15307; Sigma Aldrich; Milan, Italy) and then killed with a lethal dose of urethane (2 g/kg; #94300; Sigma Aldrich; Milan, Italy). Considering that the STZ treatment lasted for 3 months, we used 2-month-old rats, which are considered adults, to avoid their excessive aging.

From each animal, the testes were dissected and washed in preheated phosphate-buffered saline (PBS; P3813; Sigma Aldrich; Milan, Italy), and the left testis was immersed in Bouin’s solution (#HT10132; Sigma Aldrich; Milan, Italy), while the right ones were quickly frozen in liquid nitrogen and stored at −80°C for histological and biochemical analysis, respectively.

The experimental procedures were approved by the Animal Ethics Committee of the University of Campania “L. Vanvitelli” of Naples and by the Italian Ministry for Health (protocol number 30/2021). Animal care complied with the Italian (D.L. 116/92) and European Commission (O.J. of E.C. L358/1 18/12/86) regulations on the protection of laboratory animals. All efforts were made to reduce both the animal number and suffering during the experiments.

### Histology

The fixed testes were dehydrated in increasing ethanol concentrations before embedding in paraffin. Then, 5-µm-thick paraffin sections were stained with hematoxylin (#MHS1; Sigma Aldrich; Milan, Italy) and eosin (HT110216; Sigma Aldrich; Milan, Italy) for histological evaluation. Slides were examined with a Leica microscope (Leica DM 2500, Leica Microsystems, Wetzlar, Germany). Photographs were taken using Leica DFC320 R2 Digital Camera. For histopathological analysis, 30 seminiferous tubules/animal, for a total of 150 tubules per group, were counted.

### Evaluation of testicular superoxide dismutase and catalase activities and of thiobarbituric acid-reactive species levels

The enzymatic activities of superoxide dismutase (SOD) and catalase (CAT) were measured following the methods of Marklund and Marklund (42) and Claiborne (43), respectively. Enzymatic activity was expressed as units per milligram of protein (U/mg of protein).

Testis lysates (see “Protein extraction and western blot analysis”) were used to determine the thiobarbituric acid-reactive species (TBARS) levels, following a previous paper (44). The results were expressed as TBARS µM/µg of extracted protein. Each measurement was performed in triplicate.

### Testicular testosterone level measurement

Testosterone (T) levels were determined in the testis of both groups using a commercial kit (#582701; Cayman Chemical Company, Michigan, MI, USA) and using a previously published protocol for the sample’s treatment (45). Testicular T levels were expressed as ng/g of tissue.

### Protein extraction and western blot analysis

For total protein extraction, each testis was homogenized directly in RIPA lysis buffer (#TCL131; Hi Media Laboratories GmbH; Einhausen, Germany) containing 10 μL/mL of protease inhibitors mix (#39102; SERVA Electrophoresis GmbH; Heidelberg, Germany) and then centrifuged at 14,000 *g* for 20 min. For nuclear/cytoplasmic cell fractionation, each testis was homogenized in a buffer containing 0.5% Nonidet P40 (#85124; Thermo Fisher Scientific, Waltham, MA, USA) and incubated on ice for 5 min. The samples were then centrifuged at 500 g for 5 min. The supernatant, representing the cytoplasmic fraction, was collected and stored for subsequent analysis. The pellet containing the nuclear fraction was washed three times with the lysis buffer and then resuspended in the same buffer before further centrifugation at 17,500 *g*. The resulting pellet represents the nuclear fraction (46). Lowry method was used to determine the protein concentration.

In total, 40 μg of total/cytoplasmic/nuclear protein extracts was separated into SDS-PAGE (9%–15% polyacrylamide; #A4983; AppliChem GmbH, Darmstadt, Germany) and treated as described in Venditti et al. (47). For details concerning all the primary and secondary antibodies used, see **Supplementary Table S1**. The amount of protein was quantified using ImageJ software (version 1.53 t; National Institutes of Health, Bethesda, USA). Each western blot (WB) was performed in triplicate.

### Immunofluorescence analysis

For immunolocalization analysis, 5-µm testis sections were dewaxed, rehydrated, and processed as described by Venditti et al. (48). **Supplementary Table S1** reports the details about all the antibodies used. The slides were mounted with Vectashield + DAPI (#H-1200–10; Vector Laboratories, Peterborough, UK) for nuclear staining and then observed under an optical microscope (Leica DM 5000 B + CTR 5000; Leica Microsystems, Wetzlar, Germany) with a UV lamp. The images were analyzed and saved with IM 1000 software (version 4.7.0; Leica Microsystems, Wetzlar, Germany). Photographs were taken using the Leica DFC320 R2 digital camera. Densitometric analysis of immunofluorescence (IF) signal intensity and counting of positive cells was performed with the Fiji plugin (version 3.9.0/1.53 t) of ImageJ Software counting 30 seminiferous tubules/animal for a total of 150 tubules per group. Each IF was performed in triplicate.

### Terminal deoxynucleotidyl transferase dUTP nick end labeling assay

The apoptotic cells were identified in paraffin sections through the terminal deoxynucleotidyl transferase dUTP nick end labeling (TUNEL) assay using DeadEnd™ Fluorometric TUNEL System (#G3250; Promega Corp., Madison, WI, USA) following the manufacturer’s protocol, with little modifications (49). Briefly, before the incubation with TdT enzyme and nucleotide mix, sections were blocked with 5% BSA (#A1391; AppliChem GmbH, Darmstadt, Germany) and normal goat serum (#S26-M; Sigma Aldrich; Milan, Italy) diluted 1:5 in PBS and then treated with Peanut agglutinin (PNA) lectin, to mark the acrosome. Finally, the cell nuclei were counterstained with Vectashield + DAPI. The sections were observed with the same microscope described in Section 2.7. To determine the % of TUNEL-positive cells, 30 seminiferous tubules/animal for a total of 150 tubules per group, were counted using the Fiji plugin (version 3.9.0/1.53 t) of ImageJ Software. TUNEL assay was performed in triplicate.

### Statistical analysis

The values were compared by a Student’s t-test for between-group comparisons using Prism 8.0, GraphPad Software (San Diego, CA, United States). Values for p < 0.05 were considered statistically significant. All data were expressed as the mean ± standard error mean (SEM).

## Results

### Effects of T1D on oxidative stress

First, to verify the effectiveness of the STZ treatment and the onset of a diabetic state, glycemia and body weight were recorded. **Supplementary Figure S1A** shows that the serum glucose levels significantly increased in T1D rats compared to the control (*p* < 0.0001; **Supplementary Figure S1A**) soon after 1 week of STZ treatment. Concomitantly, a decrease in body weight was observed in T1D rats compared to the controls (*p* < 0.0001; **Supplementary Figure S1A**), particularly after 5 weeks of the treatment.

**Figure 1:**
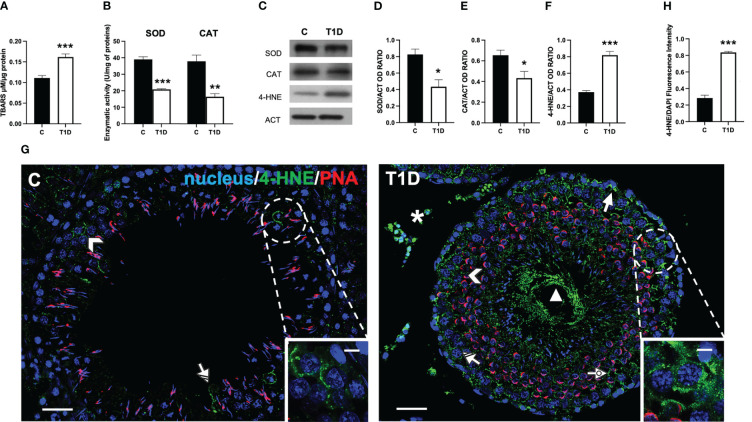
Analysis of oxidative stress parameters in control and T1D rat testis. **(A)** Testicular TBARS levels and **(B)** SOD and CAT enzymatic activities. **(C)** WB analysis of testicular SOD, CAT, and 4-HNE protein levels. **(D–F)** Histograms showing SOD, CAT, and 4-HNE relative protein levels, quantified using ImageJ and normalized to β-actin. **(G)** Testicular 4-HNE (green) immunolocalization. The slides were counterstained with PNA (red) and DAPI-fluorescent nuclear staining (blue). The images were captured at ×20 (scale bars = 20 µm) magnification and ×40 (scale bars = 10 µm) for the insets. Arrows, SPG; arrowheads, SPC; dotted arrows, SPT; triangle, SPZ tails; striped arrows, SC; asterisks, LC. **(H)** Histogram showing the quantification of 4-HNE fluorescence signal intensity. All values are expressed as means ± SEM from five animals in each group. *p < 0.05; **p < 0.01; ***p < 0.001.

As reported in **Figure 1A**, T1D induced oxidative stress since a significant increase of testicular TBARS levels, an index of lipid peroxidation, compared to the control (*p* < 0.001; **Figure 1A**) was observed. This data was supported by the analysis of the activity of the antioxidant enzymes SOD (*p* < 0.001; **Figure 1B**) and CAT (*p* < 0.01; **Figure 1B**) compared to the control. In addition, SOD (*p* < 0.05; **Figures 1C, D**) and CAT (*p* < 0.05; **Figures 1C, E**) protein levels were downregulated in the testis of T1D rats compared to the controls.

The oxidative stress induced by T1D was further analyzed by measuring the testicular levels and localization of 4-hydroxynonenal (4-HNE), one of the most biologically relevant products of lipid peroxidation that can react with proteins to form 4-HNE–protein adducts (50).

The WB analysis showed that the 4-HNE levels were significantly higher in the testes of T1D rats than those of the control group (*p* < 0.001; **Figure 1F**) The IF analysis (**Figure 1G**) showed that control testis exhibited scattered 4-HNE-positive cells, specifically spermatocytes (SPC; arrowhead and inset) and Sertoli cells (SC; striped arrow). Contrarily, in the testis of T1D rats, the signal appeared, other than in the abovementioned cells, in spermatogonia (SPG; arrow), spermatids (SPT; dotted arrow), in the tail of luminal SPZ (triangle) as well as in the interstitial LC (astersisk). Fluorescence intensity analysis showed an evident increase of 4-HNE signal in the T1D group compared to the control (*p* < 0.001; **Figure 1H**).

### Effect of T1D on apoptosis

**Figure 2:**
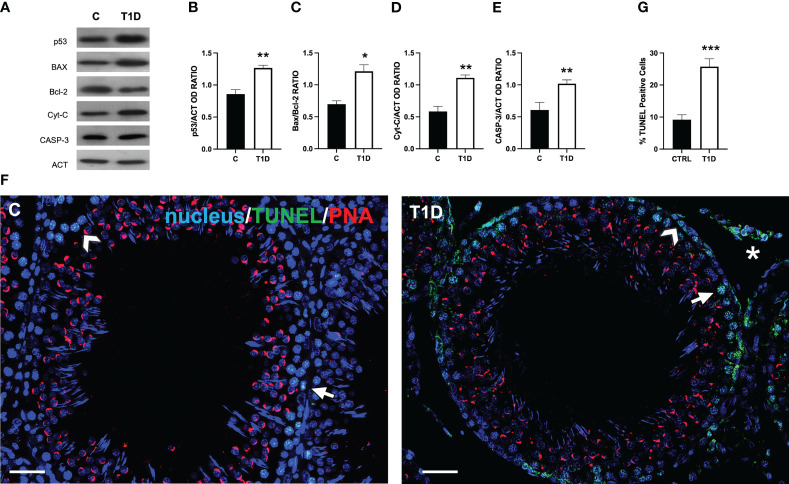
Analysis of apoptotic rate in control and T1D rat testis. **(A)** WB analysis of testicular p53, Bax, Bcl-2, cytochrome-C, and caspase-3. **(B–E)** Histograms showing the p53, Bax/Bcl-2 ratio, cytochrome-C, and caspase-3 relative protein levels quantified using ImageJ and normalized to β-actin. **(F)** Determination of apoptotic cells through the detection of TUNEL-positive cells (green). The slides were counterstained with PNA lectin (red) and with DAPI-fluorescent nuclear staining (blue). The images were captured at ×20 (scale bars = 20 µm) magnification. Arrows, SPG; arrowheads, SPC; asterisks, LC. **(G)** Histogram showing the percentage of TUNEL-positive cells. All the values are expressed as means ± SEM from five animals in each group. *p < 0.05; **p < 0.01; ***p < 0.001.

**Figure 2** shows the effect of T1D on the apoptotic rate of germ and somatic cells. The WB analysis revealed an increase in p53 (*p* < 0.01; **Figures 2A, B**), Bax/Bcl-2 ratio (*p* < 0.05; **Figures 2A, C**), cytochrome c (*p* < 0.01; **Figures 2A, D**), and caspase-3 (*p* < 0.01; **Figures 2A, E**) protein levels in the T1D group compared to the control.

To support these data, a TUNEL assay was performed (**Figure 2F**). The findings revealed the presence of scattered apoptotic cells in the control group, mainly SPG (arrow; **Figure 2F**) and SPC (arrowhead; **Figure 2F**). T1D resulted in 112% increase in the number of TUNEL-positive cells (*p* < 0.001; **Figures 2F, G**), particularly of SPG, and LC in the interstitial compartment in comparison to the control.

### Effects of T1D on the interstitial LC

**Figure 3:**
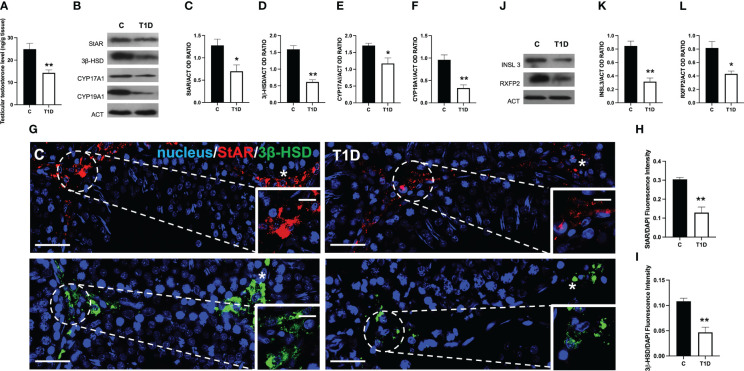
Analysis of LC activity in control and T1D rat testis. **(A)** Testicular Testosterone levels and **(B)** WB analysis of StAR, 3β-HSD, CYP17A1, and CYP19A1 protein levels. **(C–F)** Histograms showing StAR, 3β-HSD, CYP17A1, and CYP19A1 relative protein levels quantified using ImageJ and normalized to β-actin. **(G)** Testicular StAR (red) and 3β-HSD (green) immunolocalization. The slides were counterstained with DAPI-fluorescent nuclear staining (blue). The images were captured at ×20 (scale bars = 20 µm) magnification and ×40 (scale bars = 10 µm) for the insets. Asterisks, LC. **(H, I)** Histogram showing the quantification of StAR and 3β-HSD fluorescence signal intensity, respectively. **(J)** WB analysis of INSL3 and RXFP2 protein levels. **(K, L)** Histograms showing INSL3 and RXFP2 relative protein levels quantified using ImageJ and normalized to β-actin. All values are expressed as means ± SEM from five animals in each group. *p < 0.05; **p < 0.01.

Given the presence of apoptotic LC in the interstitial compartment of T1D rat testis, steroidogenesis was evaluated. **Figure 3** shows that the testicular T levels in T1D rats were significantly reduced by about 54% compared to the controls (*p* < 0.01; **Figure 3A**). The effects of T1D on testicular steroidogenesis was also evaluated by analyzing the protein levels of steroidogenic acute regulatory protein (StAR), 3β-hydroxysteroid dehydrogenase (3β-HSD), cytochrome P450 17A1 (CYP17A1), and cytochrome P450 19A1 (CYP19A1) enzymes involved in T biosynthesis. The WB analysis confirmed that T1D altered the testicular hormonal milieu, as a decrease in StAR (*p* < 0.05; **Figures 3B, C**), 3β-HSD (*p* < 0.01; **Figures 3B, D**), CYP17A1 (*p* < 0.05; **Figures 3B, E**), and CYP19A1 (p < 0.01; **Figures 3B, F**) protein levels, compared to the control, was observed. The impact of T1D on steroidogenesis was further confirmed by IF staining of StAR and 3β-HSD, which is shown in **Figure 3G**. The two signals specifically localized into the interstitial LC (asterisk; **Figure 3G** and insets) in both groups; however, fluorescence intensity analysis showed a weaker signal for StAR and 3β-HSD in T1D (*p* < 0.01; **Figures 3H, I**) compared to the control.

Finally, since the above-mentioned results clearly showed that T1D negatively impacts steroidogenesis occurring in LC, we analyzed the protein level of INSL3, a small peptide hormone secreted by mature LC that reflects their differentiation and number in the testis of all mammals and of its receptor, RXFP2 (51). The WB analysis showed that both INSL3 (*p* < 0.01; **Figures 3J, K**) and RXFP2 (*p* < 0.05; **Figures 3J, L**) were downregulated in the T1D testis compared to the controls.

### Effect of T1D on spermatogenesis

As shown in **Supplementary Figure S2A**, the testis from control rats exhibited a normal histological organization, as evidenced by GC in all the differentiative stages, including mature SPZ filling the lumen as well as LC and integral blood vessels in the interstitial compartment. The testes from T1D rats showed an altered structure, characterized by a deficiency of connections between GC (arrowhead) and, therefore, the presence of many empty spaces between them; in addition, the interstitial tissue was also affected since it appeared shrunken and the LC quite dispersed (asterisk). As shown in **Supplementary Figures S2B, C**, the analysis of two morphometric parameters further supported the histological results, as the tubules’ diameter (*p* < 0.001; **Supplementary Figure S2B**), and the percentage of tubules’ lumens full of SPZ (p < 0.001; **Supplementary Figure S2C**) were lower in the T1D group than in the controls.

**Figure 4:**
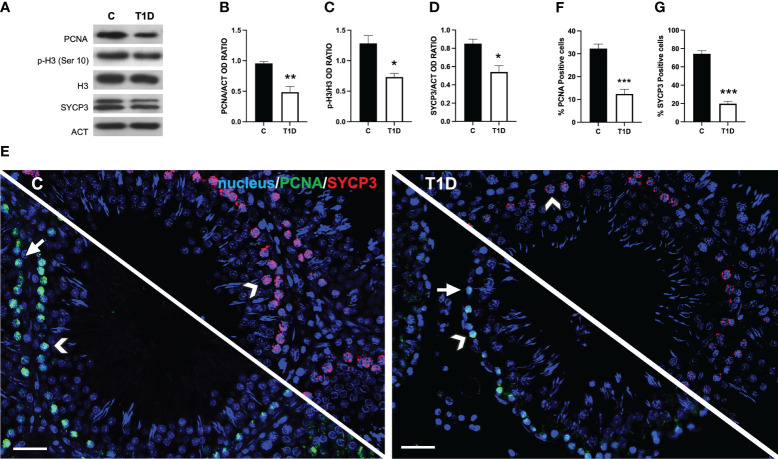
Analysis of spermatogenesis in control and T1D rat testis. **(A)** WB analysis of testicular PCNA, p-H3, H3, and SYCP3. **(B–D)** Histograms showing PCNA the p-H3/H3 ratio and SYCP3 relative protein levels quantified using ImageJ and normalized to β-actin. **(E)** Testicular PCNA (green) and SYCP3 (red) immunolocalization. The slides were counterstained with DAPI-fluorescent nuclear staining (blue). The images were captured at ×20 magnification (scale bars = 20 µm). Arrows, SPG; arrowheads, SPC. **(F, G)** Histograms showing the percentage of PCNA- and SYCP3-positive cells, respectively. All the values are expressed as means ± SEM from five animals in each group. *p < 0.05; **p < 0.01; ***p < 0.001.

To assess the effects of T1D on spermatogenesis, the protein levels of proliferating cell nuclear antigen (PCNA), phospho-histone H3 (p-H3), and synaptonemal complex protein 3 (SYCP3) were examined (**Figures 4A-D**). T1D provoked a significant decrease in PCNA (*p* < 0.01; **Figures 4A, B**), p-H3 (*p* < 0.05; **Figures 4A, C**), and SYCP3 (*p* < 0.05; **Figures 4A, D**) protein levels compared to the controls.

Data coming from IF labeling of PCNA (green panel; **Figure 4E**) and SYCP3 (red panel; **Figure 4E**) showed a PCNA-specific localization in the SPG (arrows) and SPC (arrowheads) in the testis of both groups; however, in T1D, a decrease of approximately 88% in PCNA-positive cells (*p* < 0.001; **Figure 4F**) was observed. Concerning SYCP3, it localized in the SPC nucleus (arrowheads; **Figure 4E**), and the percentage of SYCP3-positive cells decreased by 115% in the T1D group compared to the control (*p* < 0.001; **Figure 4G**).

### Effect of T1D on BTB integrity markers

**Figure 5:**
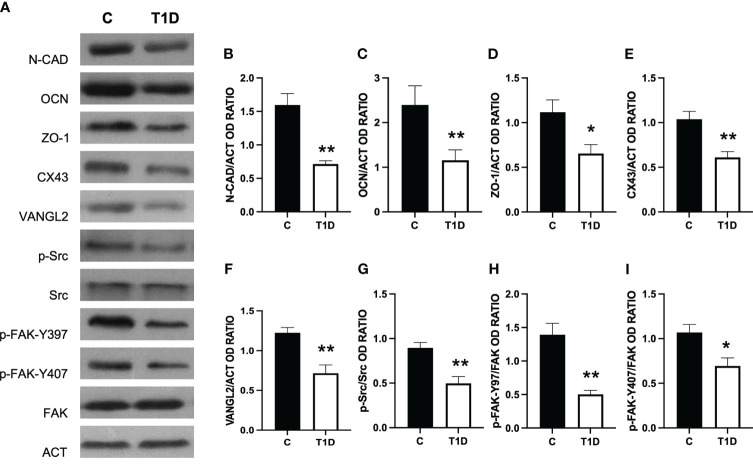
Analysis of blood–testis barrier markers in control and T1D rat testis. **(A)** WB analysis of testicular N-CAD, OCN, ZO-1, CX43, VANGL2, p-Src, Src, p-FAK-Y397, p-FAK-Y407, and FAK in the testes of T1D animals. **(B–I)** Histograms showing N-CAD, OCN, ZO-1, CX43, and VANGL2 relative protein levels and p-Src/Src, p-FAK-Y39/FAK, and p-FAK-Y407/FAK ratios quantified using ImageJ and normalized to β-actin. All the values are expressed as means ± SEM from five animals in each group. *p < 0.05; **p < 0.01.

**Figure 6:**
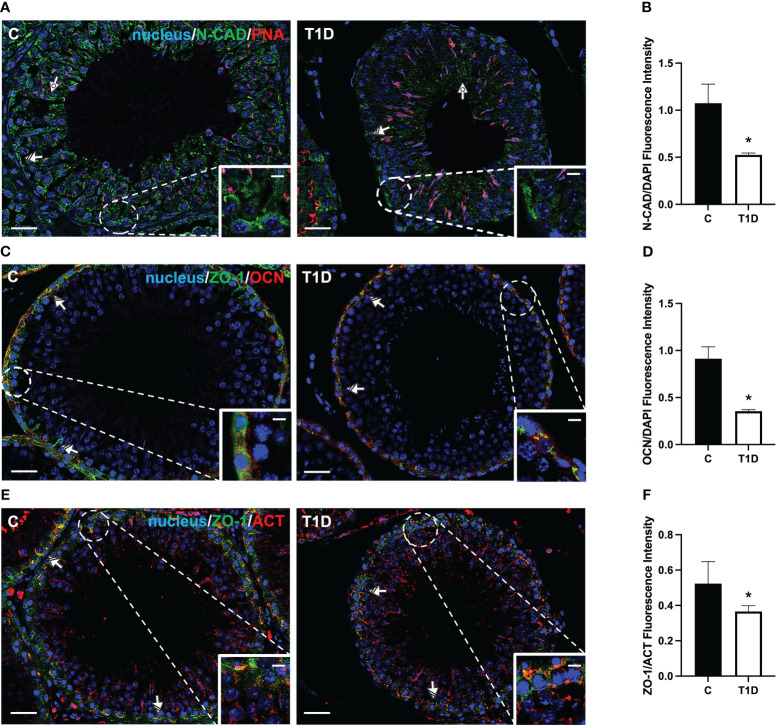
Immunofluorescent analysis of N-CAD, ZO-1, and OCN in control and T1D rat testis. **(A)** Testicular N-CAD (green) immunolocalization. The slides were counterstained with PNA lectin (red) and DAPI-fluorescent nuclear staining (blue). **(C)** Testicular ZO-1 (green) and OCN (red) immunolocalization. **(E)** Testicular ZO-1 (green) and β-actin (red) immunolocalization. All the slides were counterstained with DAPI-fluorescent nuclear staining (blue). All the images were captured at ×20 (scale bars = 20 µm) magnification and ×40 (scale bars = 10 µm) for the insets. Dotted arrows, SPT; striped arrows, SC. **(B, D, F)** Histograms showing the quantification of N-CAD, OCN, and ZO-1 fluorescence signal intensity, respectively. All the values are expressed as means ± SEM from five animals in each group. *p < 0.05.

**Figure 7:**
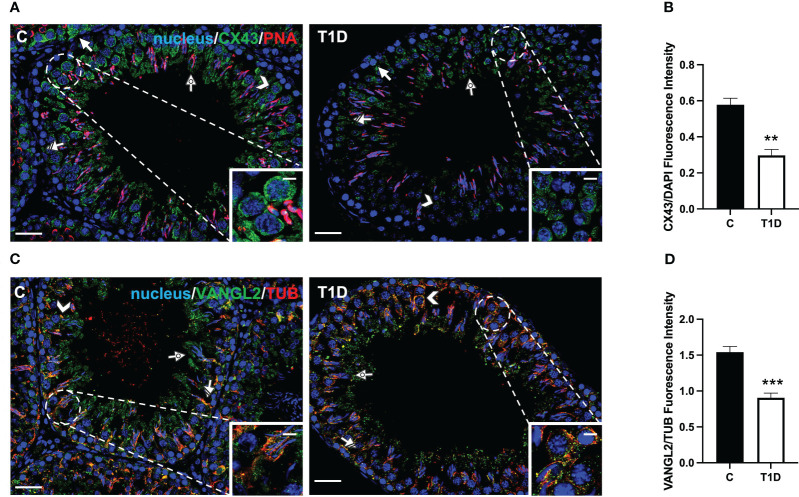
Immunofluorescent analysis of CX43 and VANGL2 in control and T1D rat testis. **(A)** Testicular CX43 (green) immunolocalization; the slides were counterstained with PNA lectin. **(C)** Testicular VANGL2 (green) and α-tubulin (red) immunolocalization. All the slides were counterstained with DAPI-fluorescent nuclear staining (blue). All the images were captured at ×20 (scale bars = 20 µm) magnification and ×40 (scale bars = 10 µm) for the insets. Arrows, SPG; arrowheads, SPC; dotted arrows, SPT; striped arrows, SC. **(B, D)** Histograms showing the quantification of CX43 and VANGL2 fluorescence signal intensity, respectively. All the values are expressed as means ± SEM from five animals in each group. **p < 0.01; ***p < 0.001.

T1D provoked considerable alterations in the BTB at both structural and regulatory proteins compared to control groups (**Figures 5**–**7**). Indeed T1D induced a significant reduction in the protein levels of N-cadherin (N-CAD; *p* < 0.01; **Figures 5A, B**), occludin (OCN; *p* < 0.01; **Figures 5A, C**), zonula occludens-1 (ZO-1; *p* < 0.05; **Figures 5A, D**), connexin 43 (CX43; *p* < 0.01; **Figures 5A, E**), and Van Gogh-Like 2 (VANGL2; *p* < 0.01; **Figures 5A, F**) as well as in the phosphorylation status of p-Src (*p* < 0.01; **Figures 5A, G**), p-FAK-Y397 (*p* < 0.01; **Figures 5A, H**), and p-FAK-Y407 (*p* < 0.05; **Figures 5A, I**) compared to the control.

For a more comprehensive description of the effects exerted by T1D on N-CAD, OCN, ZO-1 (**Figure 6**), CX43, and VANGL2 (**Figure 7**), an IF analysis was carried out. N-CAD, a protein participating in the formation of cell adhesion complexes in BTB (52), localized at the SC interface (striped arrow; **Figure 6A**), as well as in their cytoplasmic extensions through the luminal compartment, connected to the heads of elongated SPT (dotted arrow; **Figure 6A**). It is worth noting that, in the testis of T1D rats, the N-CAD signal appeared less intense in both the basal and luminal compartments, where it was quite diffuse (*p* < 0.05; **Figures 6A, B**).

OCN (**Figure 6C**) and ZO-1 (**Figure 6E**) are a transmembrane membrane and a peripheral protein, respectively, connecting tight junction (TJ) components to the actin cytoskeleton (53). They specifically localized in the SC cytoplasm (striped arrow; **Figures 6C, E**; insets) in the two groups; however, the signal intensity decreased in the T1D rats (*p* < 0.05; **Figures 6D, F**) compared to the control.

CX43 is the principal testicular gap–junction protein allowing for intracellular communication (54). IF data showed that it localized in both GC, particularly in SPG (arrows; **Figure 7A**), and SPC (arrowheads; **Figure 7A**; insets), granting their synchronous differentiation, as well as in the basal cytoplasm of adjacent SC (striped arrow; **Figure 7A**) and in their protrusions surrounding the elongating SPT (dotted arrows; **Figure 7A**). T1D provoked an evident reduction in the fluorescent intensity in SC and GC compared to the control (*p* < 0.01; **Figure 7B**).

Lastly, VANGL2, a transmembrane protein belonging to the planar cell polarity family, controls the temporal and spatial cytoarchitecture at the basal and apical ectoplasmic specialization (ES) (55, 56). In the control testis, VANGL2 is expressed in SPC (arrowheads; **Figure 7C**) and, in co-localization with tubulin, in the SC cytoplasm (striped arrows; **Figure 7C**), and their distinct protrusions were extending toward the lumen and surrounding the SPT/SPZ heads (dotted arrows; **Figure 7C**, inset). In the T1D group, though VANGL2 localized in the cell types as mentioned above (**Figure 7C**), a weaker immunofluorescent signal was observed (*p* < 0.001; **Figure 7D**). Interestingly, this may also have effects on tubulin organization since microtubules partially lose their ordered, linear structure across the seminiferous epithelium.

### Effect of T1D on testicular SIRT1/NRF2/MAPKs pathways

**Figure 8:**
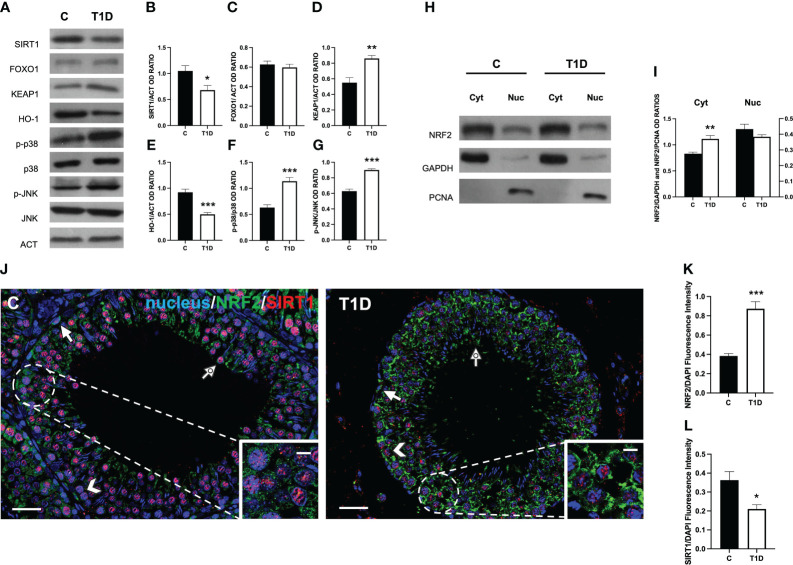
Analysis of SIRT1/NRF2/MAPKs pathways in control and T1D rat testis. **(A)** WB analysis of testicular SIRT1, FOXO1, KEAP1, HO-1, p-p38, p38, p-JNK, and JNK. **(B–G)** Histograms showing SIRT1, FOXO1, KEAP1, and HO-1 relative protein levels and p-p38/p38, and p-JNK/JNK ratios quantified using ImageJ and normalized to β-actin. **(H)** WB analysis of testicular NRF2 protein levels in cytoplasmic (Cyt) and nuclear (Nuc) fractions. **(I)** Histograms showing NRF2 relative protein levels quantified using ImageJ and normalized to GAPDH or PCNA. **(J)** Testicular NRF2 (green) and SIRT1 (red) immunolocalization. The slides were counterstained with DAPI-fluorescent nuclear staining (blue). The images were captured at ×20 (scale bars = 20 µm) magnification and ×40 (scale bars = 10 µm) for the insets. Arrows, SPG; arrowheads, SPC; dotted arrows, SPT. **(K, L)** Histograms showing the quantification of NRF2 and SIRT1 fluorescence signal intensity, respectively. All the values are expressed as means ± SEM from five animals in each group. *p < 0.05; **p < 0.01; ***p < 0.001.

Considering that T1D induces testicular oxidative stress, as previously demonstrated and herein confirmed, we decided to further investigate some well-known molecular mechanisms involved in the cellular response to oxidative stress, such as the SIRT1/NRF2/MAPKs pathways (57–60). The results showed a reduction in SIRT1 protein level in T1D rat testis compared to the control (*p* < 0.05; **Figures 8A, B**); however, no significant changes in FOXO1 levels were observed (**Figures 8A, C**). Regarding KEAP1, its protein expression significantly increased in the T1D group (*p* < 0.01; **Figures 8A, D**), while those of HO-1 (*p* < 0.001; **Figures 8A, E**) decreased compared with the control. Finally, the phosphorylation status of p38 (*p* < 0.001; **Figures 8A, F**) and JNK (*p* < 0.001; **Figures 8A, G**), was upregulated in the testis of the T1D group compared to the control.

To evaluate how T1D can modulate NRF2 nuclear translocation, we proceeded with a WB analysis performed on separated cytosolic and nuclear fractions. The results showed that T1D induced an increase of NRF2 in the cytoplasmic fraction (*p* < 0.01; **Figures 8H, I**), while its levels did not change in the nuclear fraction between the two groups.

To confirm these data, double-immunostaining of SIRT1 and NRF2 in the two groups was performed. In the control testis, SIRT1 specifically localized in SPG (arrow; **Figure 8J**), SPC (arrowhead; **Figure 8J**), and SPT (dotted arrow; **Figure 8J** and insets) nucleus. As for NRF2, it was present in the cytoplasm of the same cells (**Figure 8J**). In the testis of T1D rats, the intensity of the two signals showed an opposite behavior since it was higher for NRF2 (*p* < 0.001; **Figures 8J, K**) and weaker for SIRT1 (*p* < 0.05; **Figures 8J, L**), particularly in SPC (arrowhead; **Figure 8J**) and SPG (arrow; **Figure 8J**), respectively.

### Effect of T1D on testicular inflammation and pyroptosis

**Figure 9:**
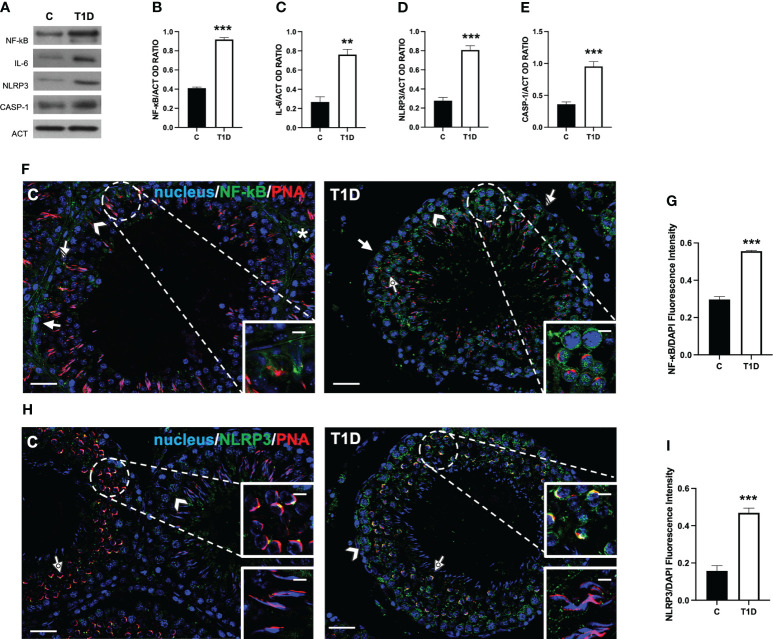
Analysis of pyroptosis in control and T1D rat testis. **(A)** WB analysis of testicular NF-κB, IL-6, NLRP3, and caspase-1. **(B–E)** Histograms showing NF-κB, IL-6, NLRP3, and caspase-1 relative protein levels quantified using ImageJ and normalized to β-actin. **(F)** Testicular NF-κB (green) immunolocalization. **(H)** Testicular NLRP3 (green) immunolocalization. All the slides were counterstained with PNA lectin (red) and with DAPI-fluorescent nuclear staining (blue). The images were captured at ×20 (scale bars = 20 µm) magnification and ×40 (scale bars = 10 µm) for the insets. Arrows, SPG; arrowheads, SPC; dotted arrows, SPT. **(G, I)** Histograms showing the quantification of NF-κB and NLRP3 fluorescence signal intensity, respectively. All the values are expressed as means ± SEM from five animals in each group. **p < 0.01; ***p < 0.001.

To assess whether a T1D induced testicular inflammation and/or pyroptosis, several markers were evaluated. The results showed an increase in NF-κB (*p* < 0.001; **Figures 9A, B**), IL-6 (*p* < 0.01; **Figures 9A, C**), NLRP3 (*p* < 0.001; **Figures 9A, D**), and caspase 1 (*p* < 0.001; **Figures 9A, E**).

As a confirmation of the above-mentioned data, an IF analysis of NF-κB (**Figure 9F**) and NLRP3 (**Figure 9H**) was carried out. In the control testis, although the intensity of the signal was quite scarce, NF-κB localized in the cytoplasm of SPG (arrow; **Figure 9F**), SPC (arrowhead; **Figure 9F**), SC (striped arrow; **Figure 9F** and insets), and interstitial LC (asterisk; **Figure 9F**). In the T1D testis, NF-κB retained its localization in the cytoplasmic compartment of the cells mentioned above, showing a more intense fluorescent signal (*p* < 0.001; **Figures 9F, G**) compared to the control. It is worth noting that a nuclear signal in SPC (dotted arrow; **Figure 9F**) was also detected in T1D animals.

As for NLRP3, we obtained the most interesting, and quite unexpected, data. Indeed in the control testis, it localized in scattered cells, as SPC (arrowhead; **Figure 9H**) and at the center of the developing acrosome of round SPT, at the cap phase of acrosome biogenesis (dotted arrow and upper inset; **Figure 9H**), while it was absent in elongating SPT at the acrosome/maturation phase (lower inset; **Figure 9H**). In the T1D testis, NLRP3 was expressed in SPC (arrowhead; **Figure 9H**) and associated with the acrosome in round SPT (dotted arrow and upper inset; **Figure 9H**), showing a more extended and fluorescent intensity (*p* < 0.001; **Figure 9I**) compared to the controls. In this case, NLRP3 was also absent in elongating SPT (lower inset; **Figure 9H**).

## Discussion

Spermatogenesis is the well-ordered sequence of distinctive cellular phases, in which SPG gives rise to SPT via mitotic and meiotic divisions that, in turn, differentiate into SPZ through spermiogenesis. Such events are at the basis of the intrinsic complexity and delicacy of spermatogenesis, which also relies on the fact that many involved molecules (i.e., ions and hormones) play a dual role, depending on their relative concentration (61, 62), and among them, ROS are of primary importance. It should be remembered that endogenous ROS are mainly generated by mitochondrial cellular respiration, and during spermatogenesis, they act as signaling molecules, activating key pathways and playing physiological roles in HPT and SPZ function (63). Conversely, excessive ROS production, together with the compromised functionality of enzymatic and non-enzymatic antioxidant defenses, drives the cellular redox status toward a pro-oxidant environment, which damages the normal structure and activity of macromolecules and organelles (64).

It is well established that T1D, together with other metabolic disorders, is a major source of oxidative stress since hyperglycemia activates “abnormal” pathways that promote ROS overproduction in many human tissues, testis included (65). In the last decades, the global incidence of T1D has increased, and although it has been usually considered to be a disease with onset during childhood, more adults than children are diagnosed each year (66).

In this paper, we first validated the successfulness of STZ treatment on testicular activity by verifying the typical features of T1D testis. Particularly, we confirmed the establishment of an oxidative stress status, as evidenced by the increased TBARS concentration, an indirect marker of lipid peroxidation, as well as the reduced activity and protein levels of the antioxidant enzymes SOD and CAT. Further evidence of this status was provided by the increase of 4-HNE, an end-product of lipid peroxidation that, reacting with proteins and DNA, can generate numerous forms of adducts (67), and due to this, it is a suitable parameter of oxidative stress. IF data also revealed that 4-HNE localized in all the cells composing the seminiferous epithelium as well as in interstitial LC. Quite expectedly, the most 4-HNE-reactive cells appeared to be the luminal SPZ since they are particularly susceptible to ROS due to inadequate antioxidant defenses and also to the presence of a high quantity of PUFA in their plasma membrane, which can be easily oxidated (68). Because of the increased oxidative stress, the results confirmed an intensified apoptotic rate in both somatic and germ cells, suggested by the increased levels of pro-apoptotic proteins as well as of an enhanced number of TUNEL-positive cells (approximately 110%) in T1D testis.

Considering that many apoptotic LC in the interstitial compartment were observed, we examined the impact of T1D on their functionality and maturity by analyzing steroidogenesis and INSL3/RXFP2, respectively. The results indicated that testicular steroidogenesis was compromised, as evidenced by the reduced testicular T levels and impaired expression of several enzymes involved in its biosynthesis. It is worth remembering that INSL3 is a hormone specifically produced and secreted by LC, uncontrolled by the HPT axis, that provides information on their differentiation status and number (51); conversely, RXFP2 is INSL3’s receptor and is expressed mainly in testicular post-meiotic cells (69). In addition, several studies demonstrated that INSL3, by activating RXFP2, may regulate GC turnover and apoptosis, especially when spermatogenesis is under stress conditions (70), as that induced by T1D.

As discussed above, both T and INSL3 regulate indispensable steps for the normal progression of spermatogenesis; thus, their impaired levels (together with the induced apoptosis) are reflected by the reduced expression of mitotic (p-H3; PCNA) and meiotic (SYCP3) markers as well as by the reduction of 88% and 115% PCNA- and SYPC3-positive cells, respectively. The combined data led us to hypothesize that T1D, through the induction of oxidative stress and disturbance of LC activity, reduces their ability to produce T and INSL3, which has a direct effect on spermatogenesis, as evidenced by the reduction in kinetic/dynamic activity of GC.

It is well known that during the progression leading SPG to differentiate into SPZ, which will be released into the tubules’ lumen, cells are actively transported thanks to their intimate connection with SC, the somatic component of the seminiferous epithelium. Indeed SC creates a unique structure, the BTB, which divides the epithelium into two compartments: basal, where SPG and preleptotene SPC are located, and an apical one, in which all the other cell types reside. In addition, BTB is responsible for the establishment of an immune-privileged environment, allowing the testes to tolerate the antigenic factors expressed by the SPZ, protecting them from systemic immune attack (71). BTB is an extremely dynamic structure, placed between adjacent SC and SC–GC, and is based on structural, scaffolding, and signaling proteins that work coordinately through cycles of phosphorylation/de-phosphorylation, endocytosis of membrane proteins, and their recycling to ensure the correct movement of GC (53).

Herein we confirmed that in the testis of T1D rats, the protein levels of N-CAD, ZO-1, OCN, and CX43 were reduced (72, 73). However, to our knowledge, this is the first report showing that T1D also affects the testicular levels of VANGL2, a protein located at the apical ES, as well as the activation of Src and FAK. VANGL2 regulates the spatial and temporal organization of actin microfilaments and microtubules, playing a significant role in controlling BTB integrity and SPT transit toward the lumen (56). IF data revealed an altered distribution of VANGL2 as well as that of tubulin in the SC cytoplasmic protrusions, strongly suggesting that T1D may also act on the cytoskeletal organization of testicular cells.

Regarding Src and FAK, their tandem phosphorylation allows interaction with other BTB components, such as OCN and ZO-1, regulating the GC transit and maintaining BTB integrity. Thus, as also previously demonstrated by other papers in T1D rodents (72, 73), we confirmed that BTB is compromised, emphasizing that its stability is fundamental for proper spermatogenesis. However, as a limitation of this study, these are indirect data, and an *in vivo* BTB integrity assay would offer direct evidence, solidifying the claim.

For a broader picture of the underlying molecular pathways of the T1D effects on testicular activity, we analyzed the contribution of some key factors notoriously involved in the oxidative stress response, namely, SIRT1, NRF2, and the MAPKs p38/JNK. The transcription factor NRF2 represents the central hub of this system since it is an adaptive cellular defense response to different stresses, including oxidative and metabolic ones (74). In normal conditions, NRF2 is “sequestered” in the cytoplasm by KEAP1, a ubiquitin E3 ligase, which promotes its degradation through ubiquitination; when an oxidative insult hits cells, the increased ROS production leads to NRF2 accumulation, its nuclear translocation, and the activation of ARE-dependent genes, including the antioxidant enzymes HO-1 and SOD (74). The regulation of NRF2 activity is multifaced since it has been demonstrated by previous reports that other events may be involved, such as NRF2 deacetylation, promoted by SIRT1 (75), and/or its phosphorylation, induced by MAPKs (76). Herein we found that T1D downregulated testicular SIRT1 expression, while KEAP1 protein level and the phosphorylation status of p38 and JNK were upregulated. This, in turn, may lead to the deregulation of NRF2 signaling that, not translocating toward the nucleus, is not able to activate the cellular antioxidant defenses.

Worthy of note is that NRF2 seems to be a vital regulator of the molecular mechanisms that cause male infertility. In fact, several studies suggest that the function of NRF2 plays a crucial role in protecting spermatogenesis when an excessive amount of ROS is produced (77) as in the case of exposure to environmental pollutants (78, 79), aging (80), and metabolic disorder (17, 81). This protection, in turn, preserves specific sperm functions, particularly motility, which could otherwise be compromised under conditions of increased ROS exposure. This point is of interest since many intervention strategies, aimed to ameliorate/counteract prooxidant stimuli posed by different etiologies (such as disease or exposure to environmental pollutants), have as a target just the activation of the SIRT1/NRF2 pathways (77, 82).

All metabolic disorders, T1D included, are characterized by an intimate relationship between oxidative stress and inflammation: via the generation of a vicious cycle, oxidative stress stimulates inflammatory pathways, which, in turn, enhance ROS overproduction (83, 84). Due to this, we analyzed whether the induction of testicular inflammation was elicited by T1D. As well known, the principal actor involved in inflammation activation is transcription factor NF-κB since most of the genes encoding for pro-inflammatory cytokines possess, in their promoter/enhancer regions, NF-κB-binding sites (85). Here we found that T1D increased the testicular expression of both NF-κB and the pro-inflammatory cytokine IL-6, thus confirming the activation of the inflammatory pathway.

NLRP3 is a gene under NF-κB transcriptional control, the main component of the inflammasome (86). Inflammasome is a cytosolic, multiprotein complex which, once assembled, elicits the proteolytic cleavage and then activation of procaspase-1 into active caspase 1 that also induces the activation of other proinflammatory mediators, such as IL-1b, or a specific proinflammatory cell death defined as pyroptosis (87). Our results showed that T1D also activated inflammasome, as well as testicular pyroptosis, probably in response to enhanced oxidative stress and inflammation, contributing to apoptosis of germ and somatic cells. These results are partially in line with other reports showing that oxidative status, induced by obesity (88), varicocele (89), and exposure to toxicants (90, 91), enhanced the expression of NLRP3 and of the downstream pathways, leading to testicular pyroptosis, inflammation, and, ultimately, to testicular dysfunction. However, in most of those studies, the main cellular source of NLRP3 production appeared to be the SC, and thus its correlation with perturbation with BTB integrity has been hypothesized; this point was supported by the fact that inhibiting NLRP3 pathway increased the expression of TJ components, consequently ameliorating the BTB integrity (92, 93).

Interestingly, although we could not detect any signal in SC cytoplasm, IF data revealed a peculiar NLRP3 localization in both control and T1D testis, indeed it was associated with the developing acrosome system in the control, assuming a dot-shaped conformation in round SPT while, in T1D, its extension and signal intensity were increased, particularly in SPT, where a clear nuclear NF-κB signal was also observed. Considering that acrosome biogenesis involves the generation of Golgi-derived vesicles (94) and that the Golgi apparatus seems fundamental for the recruitment and activation of NLRP3 (95, 96), we hypothesize that NLRP3 may also be somehow involved in the formation of this organelle. However, although fascinating, this hypothesis needs more in-depth investigation, and in-progress studies are focused just to clarify this aspect.

## Conclusion

This study confirms that STZ-induced T1D negatively impacts rat testicular activity. We verified that T1D, by inducing oxidative stress, produces damage in both the somatic and germinal compartments, as evidenced by the alteration of several parameters, such as steroidogenesis, spermatogenesis, and BTB integrity. Mechanistically, these effects may be related to the dysregulation of the SIRT1/NRF2/MAPKs pathways and, particularly, by the increased cytoplasmic retention of NRF2, which is not able to activate adequate antioxidant defenses. In addition, induction of testicular inflammation and pyroptosis was also demonstrated, as highlighted by the increased levels of some markers, such as NF-κB and NLRP3. Finally, the localization of NLRP3 in the acrosome region suggested its peculiar involvement in its biogenesis. The combined data encourages further studies to confirm not only this aspect but also on the development of strategies to be used in preventing/mitigating the effects of T1D and other metabolic disorders on human male fertility.

## Data availability statement

The raw data supporting the conclusions of this article will be made available by the authors, without undue reservation.

## Ethics statement

The experimental procedures were approved by the Animal Ethics Committee of the University of Campania “L. Vanvitelli” of Naples and by the Italian Ministry for Health (protocol number 30/2021). The study was conducted in accordance with the local legislation and institutional requirements.

## Author contributions

MV: Conceptualization, Funding acquisition, Investigation, Visualization, Writing – original draft. MR: Investigation, Visualization, Writing – original draft. SB: Investigation, Methodology, Writing – original draft. AH: Investigation, Writing – original draft. AB: Investigation, Writing – original draft. SMa: Methodology, Writing – review & editing. SMi: Conceptualization, Supervision, Writing – review & editing.
