# Supplementary material for: Type 1 diabetes impairs the activity of rat testicular somatic and germ cells through NRF2/NLRP3 pathway-mediated oxidative stress
Source: Front Endocrinol (Lausanne). 2024 May 16;15:1399256. doi: 10.3389/fendo.2024.1399256 (PMC11137174; doi:10.3389/fendo.2024.1399256)
Supplement: Supplementary file 1 [file DataSheet_1.docx]

Supplementary Material


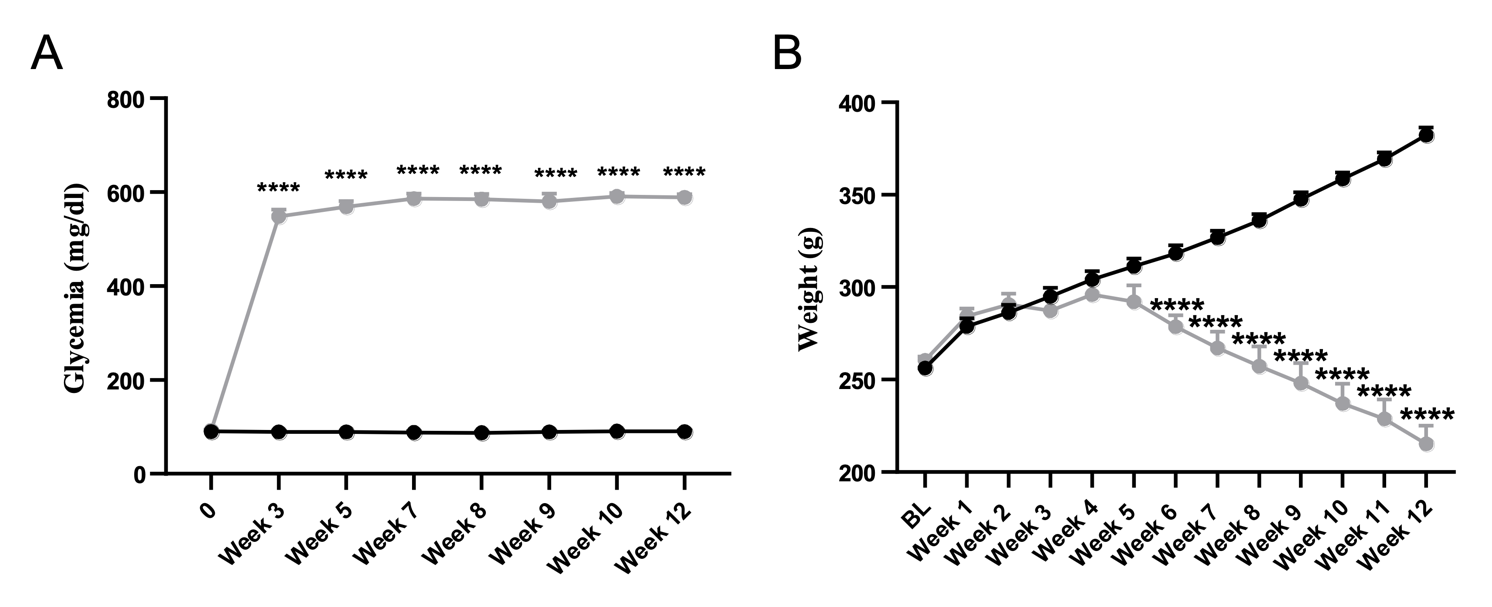


**Supplementary Figure 1.** Metabolic parameters of control and T1D rat testis. (A) Serum glucose levels, expressed as milligrams per deciliter, in control and T1D rat, recorded every week. (B) Body weight of the control and T1D animals, recorded every week. In both graphs, the black line represents control rat, while the grey ones T1D animals. All the values are expressed as means ± SEM from 5 animals in each group. **** p < 0.0001.


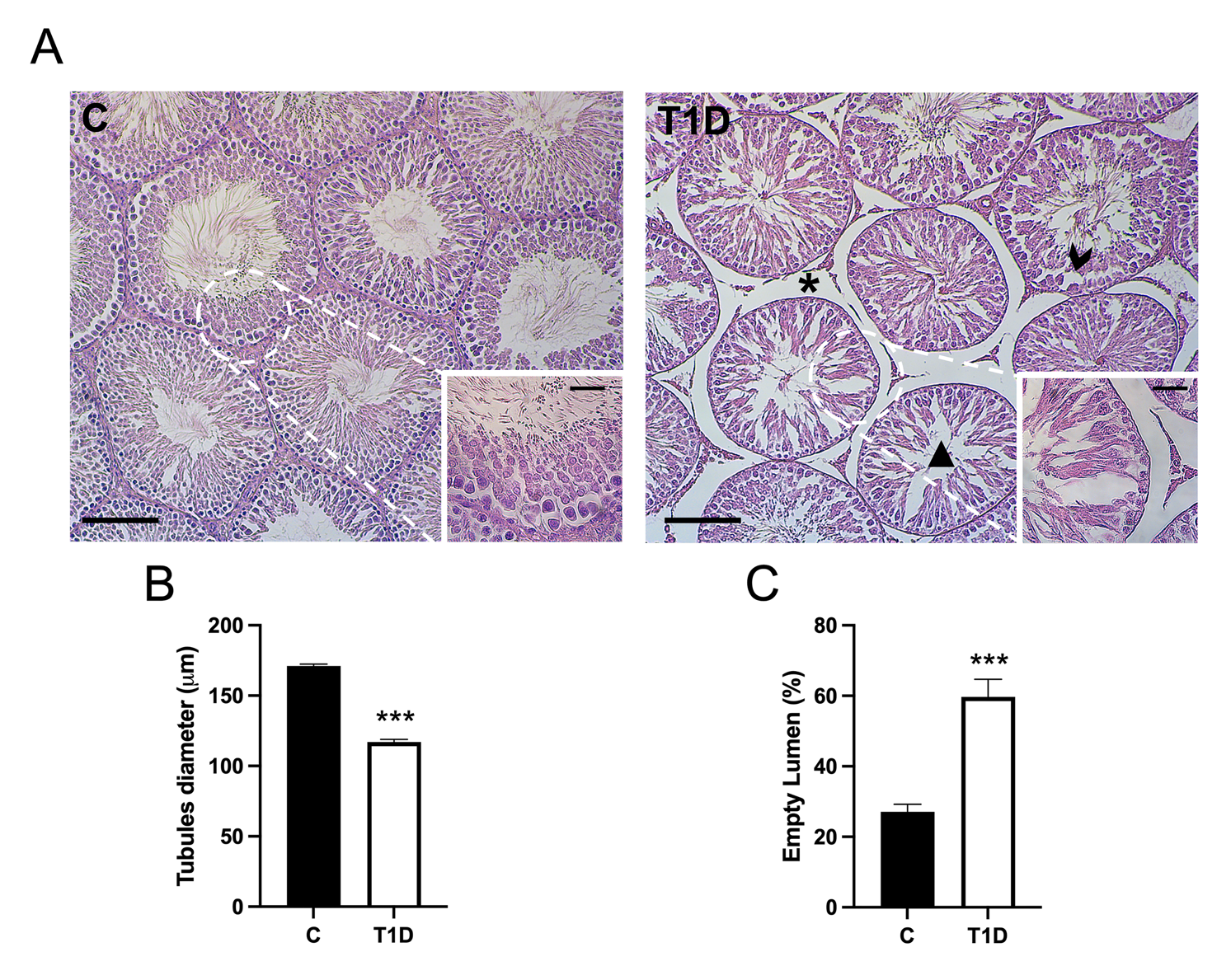


**Supplementary Figure 2.** Histological analysis of control and T1D rat testis. (A) Hematoxylin-eosin staining of rat testicular paraffin-embedded sections. The images were captured at x10 (scale bars= 50 µm) magnification and x40 (scale bars= 10 µm) for the insets. Arrowheads: empty spaces between GC; triangle: tubules lumen; asterisks: interstitial compartment. (B) Tubules diameter and (C) % of the empty lumen in control and T1D rat testis. All the values are expressed as means ± SEM from 5 animals in each group. *** p < 0.001.

**Table S1.** List of all the used antibodies.

| **Antibody** | **Molecular weight (kDa)** | **WB Dilution** | **IF Dilution** | **Source** |
| --- | --- | --- | --- | --- |
| SOD | 16 | 1:2000 | - | Cell Signaling Technology, Danvers, MA, USA  #65776 |
| CAT | 60 | 1:1000 | - | Cell Signaling Technology, Danvers, MA, USA  #214097 |
| 4-HNE | 66 | 1:1000 | 1:100 | Thermo Fisher Scientific, Waltham, MA, USA  #BS-6313R |
| p53 | 43 | 1:500 | - | Elabscience Biotechnology, Wuhan, China  #E-AB-32469 |
| BAX | 21 | 1:750 | - | Elabscience Biotechnology, Wuhan, China  #E-AB-13814 |
| Bcl-2 | 22-26 | 1:750 | - | Elabscience Biotechnology, Wuhan, China  #E-AB-60012 |
| Cyt-C | 21 | 1:700 | - | Elabscience Biotechnology, Wuhan, China  # E-AB-64633 |
| Caspase-3 | 17 | 1:700 | - | Elabscience Biotechnology, Wuhan, China  #E-AB-22115 |
| StAR | 32 | 1:700 | 1:100 | Elabscience Biotechnology, Wuhan, China  #E-AB-15419 |
| 3β-HSD | 42 | 1:700 | 1:100 | Elabscience Biotechnology, Wuhan, China  #E-AB-15112 |
| CYP17A1 | 57 | 1:1000 | - | Elabscience Biotechnology, Wuhan, China  #E-AB-60298 |
| CYP19A1 | 58 | 1:1000 | - | Santa Cruz Biotechnology, Santa Cruz, CA, USA  #sc-30086 |
| PCNA | 36 | 1:1000 | 1:100 | Sigma-Aldrich, Milan, Italy  #98825 |
| phospho-Histone H3 | 17 | 1:1000 | - | Merck Millipore, Milan, Italy  #06–570 |
| Histone H3 | 17 | 1:1000 | - | Merck Millipore, Milan, Italy  #06–755 |
| SYCP3 | 30–33 | 1:250 | 1:50 | Santa Cruz Biotechnology, Santa Cruz, CA, USA  #sc-74569 |
| N-Cadherin | 125-135 | 1:1000 | 1:100 | Abcam, Cambridge, UK  #ab18203 |
| OCN | 65 | 1:1000 | 1:100 | Thermo Fisher Scientific, Waltham, MA, USA  #33-1500 |
| ZO-1 | 200 | 1:2000 | 1:100 | Thermo Fisher Scientific, Waltham, MA, USA  #40-2200 |
| CX43 | 43 | 1:1000 | 1:100 | Elabscience Biotechnology, Wuhan, China  #E-AB-70097 |
| VANGL2 | 60 | 1:500 | 1:100 | Sigma-Aldrich, Milan, Italy  #ABN2242 |
| p-Src | 60 | 1:1000 | - | Cell Signaling Technology, Danvers, MA, USA  #2101 |
| Src | 60 | 1:1000 | - | Cell Signaling Technology, Danvers, MA, USA  #2102 |
| p-FAK-Y397 | 125 | 1:500 | - | Thermo Fisher Scientific, Waltham, MA, USA  #44-625G |
| p-FAK-Y407 | 125 | 1:500 | - | Thermo Fisher Scientific, Waltham, MA, USA  #44-650G |
| FAK | 125 | 1:1000 | - | Thermo Fisher Scientific, Waltham, MA, USA  #39-6500 |
| SIRT1 | 120 | 1:3000 | 1:100 | Abcam, Cambridge, UK  #ab110304 |
| FOXO1 | 78 | 1:1000 | - | Elabscience Biotechnology, Wuhan, China  #E-AB-31466 |
| KEAP1 | 60 | 1:700 | - | ABclonal Science, Inc. Woburn, MA, USA  #A11258-20 |
| HO-1 | 33 | 1:1500 | - | GeneTex, Irvine, CA, USA  #GTX101147 |
| p-p38 | 41 | 1:500 | - | Sigma-Aldrich, Milan, Italy  #MABS64 |
| p38 | 41 | 1:1000 | - | ABclonal Science, Inc. Woburn, MA, USA  #A14401 |
| p-JNK | 46 | 1:500 | - | Santa Cruz Biotechnology, Santa Cruz, CA, USA  #sc-12882-R |
| JNK | 46 | 1:500 | - | Elabscience Biotechnology, Wuhan, China  #E-AB-60070 |
| NRF2 | 68 | 1:2000 | 1:100 | GeneTex, Irvine, CA, USA  #GTX103322 |
| GAPDH | 37 | 1:5000 | - | Cell Signaling Technology, Danvers, MA, USA  #97166 |
| NFkB p65 | 100 | 1:1000 | 1:100 | Elabscience Biotechnology, Wuhan, China  #E-AB-65807 |
| IL-6 | 23 | 1:1000 | - | Elabscience Biotechnology, Wuhan, China  #E-AB-30095 |
| NLRP3 | 118 | 1:2000 | 1:100 | GeneTex, Irvine, CA, USA  #GTX00763 |
| Caspase-1 | 25 | 1:1000 | 48 | Cell Signaling Technology, Danvers, MA, USA  #83383 |
| β-Actin | 42 | 1:5000 | 1:100 | Elabscience Biotechnology, Wuhan, China  #E-AB-20031 |
| α-Tubulin | 52 | - | 1:100 | Elabscience Biotechnology, Wuhan, China  #E-AB-20036 |
| Goat anti-rabbit IgG HRP | - | 1:5000 | - | Vector Laboratories, Burlingame, CA, USA  #PI-1000 |
| Goat anti-mouse IgG HRP | - | 1:5000 | - | BioActs, Namdong-gu, Incheon, Korea  #RSA1122 |
| Goat anti-rabbit  Alexa Fluor 488 | - | - | 1:500 | Thermo Fisher Scientific, Waltham, Ma, USA  #A32731 |
| Goat anti-mouse  CF™ 568 | - | - | 1:250 | Sigma-Aldrich, Milan, Italy  #SAB4600082 |
| PNA lectin   Alexa Fluor 568 | - | - | 1:50 | Thermo Fisher Scientific, Waltham, Ma, USA  #L32458 |
